# Adaptation of a trauma-informed intervention for youth involved in the legal system

**DOI:** 10.21203/rs.3.rs-2596631/v1

**Published:** 2023-03-01

**Authors:** Erin Becker Razuri, Yang Yang, Elaine Tinius, Danica Kalling Knight

**Affiliations:** Texas Christian University; Texas Christian University; Texas Christian University; Texas Christian University

**Keywords:** Trust-based Relational Intervention (TBRI), youth in the legal system, adaptation, ADAPT-ITT, trauma intervention, juvenile justice

## Abstract

**Background:**

Youth in the legal system (YILS) have high rates of trauma exposure, which are associated with increased risk of behavioral health needs (e.g., substance-use problems) and recidivism. Research suggests that a trauma-focused therapeutic approach can improve outcomes for YILS, but few evidence-based interventions (EBIs) are designed with justice-involved youth in mind. Consequently, implementing trauma-informed EBIs within juvenile justice (JJ) systems is challenging. The current paper describes the systematic adaptation of Trust-based Relational Intervention (TBRI) as a substance use prevention intervention for YILS and their caregivers.

**Methods:**

The current study utilized a methodology based on the ADAPT-ITT framework to adapt TBRI Caregiver Training, an evidence-based, trauma-informed intervention program. Phases of adaptation included (1) *Assessment*, (2) *Decision*, (3) *Prototype Development*, and (4) *Testing and Integration*. The adaptation process explored contextual factors (e.g., systems, facilities, and staff) and the needs of the new target population (i.e., YILS and their caregivers). Adaptations were made to both content (e.g., terminology and activities) and structure (e.g., session duration and delivery setting) in an iterative process with input from participants from the target population, key stakeholders, and content experts.

**Results:**

The systematic adaptation of the intervention model resulted in a two-phase, four-component intervention package that can be implemented in juvenile justice settings as part of youth reentry services. The primary intervention, delivered while youth are in residential facilities, includes the *TBRI Caregiver Curriculum, TBRI Youth & Young Adult Curriculum, and TBRI Nurture Groups*. The secondary intervention, delivered after youth transition home, includes the *TBRI Family Coaching Curriculum*.

**Conclusions:**

Utilizing an implementation science framework to guide adaptation has implications for developing accessible, culturally relevant, and contextually-appropriate interventions. Accounting for contextual factors and population needs can improve the fit of EBIs in juvenile justice, facilitating uptake and ultimately improving outcomes for youth.

**Trial registration:**

ClinicalTrials.gov Identifier: NCT04678960

## Background

The high prevalence of trauma among youth in the legal system (YILS) is well-documented, with trauma experienced by upwards of 75% of youth entering the legal system [1, 2]. Adverse childhood experiences (ACEs; e.g., abuse, neglect, household dysfunction) are associated with increased risk for behavioral health needs (e.g., substance use problems) [3] and further involvement with the justice system (e.g., recidivism) [4]. Research suggests that addressing behaviors associated with trauma can improve a range of outcomes for YILS, perhaps by addressing underlying psychological and physiological needs [5]. Indeed, there is robust evidence indicating that therapeutic approaches to youth justice (i.e., programs that attempt to bring about behavioral change by focusing on relationships, personal insight, and skill building) [6] are notably more effective at reducing recidivism than programs oriented towards control (i.e., programs that attempt to suppress delinquency, such as through discipline, deterrence, and surveillance) [7, 8].

Trauma-informed treatment would appear to offer a promising approach to intervention in juvenile justice (JJ) [5]. However, there is a greater implementation gap in the JJ system in comparison to other human service systems (e.g., behavioral health, education, public health) [9]. Few EBIs are designed for JJ populations, and even fewer have data to suggest that they can be implemented with fidelity at scale [10]. Only one-third of community supervision agencies and behavioral health providers in the JJ system report offering such programs [11] and the JJ system is rife with resistance to evidence-based practice and therapeutic intervention [6]. Tailoring existing EBIs to fit JJ contexts can be an efficient and effective implementation strategy to meet a critical need without compromising outcomes.

## Trust-based Relational Intervention^®^ (TBRI^®^)

TBRI is a trauma-informed, attachment-based approach to caring for children and youth who have experienced early adversity and potentially traumatic stress [12]. Consistent with the three pillars of trauma-informed care [13], TBRI is based on three harmonious and synergistic principles: *Connecting, Empowering*, and *Correcting*. As part of the Leveraging Safe Adults (LeSA), TBRI was adapted for JJ-involved families [14]. Given that most youth discharged from secure residential facilities return to existing family/living environments, equipping adults who are responsible for their care with effective tools and support is critical and represents an urgent and potentially transformative prevention/intervention opportunity. LeSA’s primary aim is to leverage existing relationships with caregivers to support youth transitioning out of JJ facilities. Caregivers (biological, adoptive, and foster parents) and guardians (e.g., grandparents) are trained to be “safe adults” for their youth, by building trust, practicing authentic communication, developing boundaries, and setting realistic expectations in order to identify and address their youth’s needs proactively and effectively. Through their relationships with safe adults, youth learn and practice self-regulation, enabling them to more effectively refrain from opioid use, other SU, and other risky activities.

The LeSA project utilized an evidence-based, standardized TBRI intervention protocol, the TBRI Caregiver Training Package [15]. Training is delivered by TBRI Practitioners to caregivers or to staff in child-serving settings (e.g., residential care staff, mental health professionals, case workers). Caregiver Training is designed to move from theory into practical applications by giving participants a knowledge base, then providing them with real-life strategies and tools they can use with children and youth. Caregiver Training, originally developed for adoptive parents of school-age children, is now used to train caregivers in an ever-expanding range of service settings and with increasingly diverse populations [16, 17].

### Approach to Adaptation

Adaptation, the process of making an evidence-based intervention fit a specific or new use or situation [18], is increasingly recognized within implementation science as an important strategy to accelerate the uptake of EBI into standards of care and encourage research/practice collaborations that address the particular needs of diverse communities and the unique characteristics of various service settings [19]. Thoughtful adaptation processes can promote successful implementation within justice settings by helping to ensure that interventions are accessible, effective, contextually appropriate, and culturally relevant.

Within implementation science, there is growing interest in a systematic approach to identifying and organizing the types of modifications and adaptations made to improve the fit of an EBI [20]. For the purposes of the current study, adaptations are organized into two broad categories: adaptations to *content* and adaptations to *structure*. Content adaptations refer to adaptations to the specific practices, techniques, procedures, or other intervention elements that make up the EBI protocol and characterize facilitator-participant interactions, including refining, adding, removing, condensing, and extending intervention elements [20, 21]. Structural adaptations refer to the procedures and processes that organize and inform the delivery of intervention content, including session length, program duration, setting, and facilitator. Adaptations can also be organized by contextual factors, from adaptations made to meet the needs of individuals and groups in the new target population (e.g., language, caretaking practices, and social norms) [22]; to organizational and staff factors (e.g., organizational culture and climate) [23]; to systemic and structural factors (e.g., policies, funding mandates) [18]. Given the complex interplay of systemic, site, and individual factors that can make implementation in JJ systems particularly challenging [6, 7], systematic, comprehensive approaches to adaptation are critical.

## Current Study

The purpose of the current study is to describe the process and outcomes associated with the systematic adaptation of TBRI as a family-centered intervention to prevent substance use among YILS. The aim was to balance fidelity and flexibility in the revised curriculum through an iterative, data-driven adaptation process, with input from stakeholders, content experts, and members of the new target population. The detailed methodological approach and comprehensive description of the resulting intervention package provide a concrete illustration of planned adaptation efforts which are critical to successful implementation. Ultimately, such efforts would promote the successful implementation of trauma-informed care within JJ systems.

## Methods

The current study utilized a methodology based on ADAPT-ITT [24], a phased, systematic process for adapting evidence-based interventions. The ADAPT-ITT model was selected as a guiding framework for a number of notable features, including (a) the direct involvement of members of the target population and key stakeholders throughout the adaptation process, (b) incorporation of diverse measures, including qualitative and quantitative assessments, (c) promotion of a balance between fidelity and flexibility, and (d) facilitation of a documented adaptation plan. The current paper focuses on the early phases of the adaptation process, from the initial assessment of needs to development of the final intervention product. Content experts involved in the adaptation process had extensive clinical experiences with children and youth of all ages and had implemented the TBRI Caregiver Training in diverse settings (e.g., with foster families and JJ-involved families, in clinical settings) and external stakeholders, including direct care and administrative staff in the JJ system. The research project was approved by the university Institutional Review Board and all participants provided written informed consent.

### Phase 1: Assessment

The adaptation process began by assessing the needs of the new target population and collecting information about the implementation context. The original adaptation plan developed by the principal investigator involved surface-level adaptations to the content and structure of TBRI Caregiver Training and development of a companion curriculum to guide in-home family coaching and support youth following discharge from juvenile facilities. Feedback from early conversations with JJ stakeholders and topic experts contributed to four initial targets for adaptation: (a) revise language, activities, and other surface-level content to be more relevant for youth and young adults, (b) revise language to be more inclusive of diverse family systems, (c) shorten overall program duration, and (d) modify structural elements of Caregiver Training for in-home delivery consistent with home coaching programs.

Focus groups were conducted to obtain inputs on two areas, including (a) youth reentry processes and family environment and (b) TBRI implementation in JJ settings (see Table 1).

Focus groups were conducted with a total of 59 staff members from several JJ partners, including 1 state JJ organization, 4 county juvenile probation departments that operated secure residential facilities and community-based supervision, 2 private facilities providing services to post-adjudicated youth, and 2 state-owned facilities providing services to local counties. Staff at eligible research sites were selected based on site recommendations and invited to participate by the university research team. All focus group participants provided informed consent. Focus groups were conducted on-site at facilities, with the exception of one interview that was converted to virtual to accommodate COVID-19 protocols. Focus groups were facilitated by the principal investigator and followed a semi-structured interview protocol. Conversations were audio recorded and transcribed for subsequent analysis.

#### Reentry Processes and Family Environments

Eight focus groups were conducted with participants from six JJ sites in two states participating in the LeSA project. Questions focused on reentry processes and family environments for YILS (e.g., *Please describe how decisions about home/caregiver placement are determined*.) Four sites had previous experience with TBRI. Interview length ranged from 1 to 2 hours, with a total interview time of 14.5 hours. Participants included 36 staff members representing 6 sites, with some staff participating in more than one interview. The number of staff participating in each interview ranged from 1 to 12. Positions of participating staff included direct care (e.g., case managers) and leadership (e.g., Chief Officer).

#### TBRI Implementation in JJ Settings

Additional focus group interviews were conducted with participants from a subset of four sites that had begun implementing TBRI in their organizations and had experience trying to fit existing TBRI language, activities, and strategies with their population. Questions focused on experiences using TBRI components, suggestions for adapting TBRI for older adolescents and young adults, and input regarding family recruitment for TBRI interventions (e.g., *In what ways have you modified the terminology and recommended strategies to better meet the needs of the youth you serve*?). Interview length ranged from 2.5 to 6 hours, with a total interview time of 13 hours. Participants included 23 staff from four agencies. Participants included staff who had directly adopted TBRI components in youth service provision (e.g., substance use counselor) and leadership supervising the local implementation team (e.g., director of reentry-services).

### Phase 2: Decision

The second phase of adaptation involved making decisions regarding what modifications to make to the existing intervention. Decision-making was informed by close examination of focus group content and input from content experts (people with experience developing curricula and practitioners with experience applying TBRI with adolescents). Project leadership (principal investigator, project director) established an EBI adaptation workgroup (i.e., aforementioned content experts) charged with reviewing focus group data, identifying curriculum elements in need of revision, and recommending suggested changes to content, structure, language, and activities. Plans were approved by project leadership.

### Phase 3: Prototype Development

Phase three involved revising the EBI and developing a prototype for testing. The prototype was developed by the TBRI adaptation workgroup, which met weekly during the *Prototype Development* and *Testing and Integration* phases of adaptation. Informed by previous phases, the group identified core elements of the EBI to remain intact [25] and ascertained new needs and potential targets for adaptation. The workgroup adopted a round-robin approach with Delphi methods because the flexible and iterative format allows for gathering and consolidating multiple rounds of inputs in the target intervention [26]. Completion of the first prototype took approximately six months.

### Phase 4: Testing and Integration

Prototype development was followed by a pilot test of the adapted EBI with individuals from the new target population, referred to in the ADAPT-ITT model as theater testing. Testing was conducted at two sites, with separate groups for caregivers and youth delivered weekly, and joint caregiver/youth sessions delivered biweekly. The start dates of delivery at the sites were one week apart to allow slight adjustments based on what was learned in the first site. Pilot facilitators were members of the workgroup and TBRI Practitioners with experience training and implementing TBRI with caregivers and youth. Facilitators took notes during each session and shared feedback (what went well, what was tricky) with the workgroup on a weekly basis. Based on this information, workgroup members produced new/further-revised content and activities and integrated revisions into the prototype. This iterative process, in which feedback was documented and immediately shared with the workgroup, allowed modifications to be made quickly. Necessary changes were rapidly integrated into the curricula for testing at the second site. Less time-sensitive changes were discussed and incorporated into subsequent drafts. Integration took place across curriculum components to improve consistency. Refinements made to one session (e.g., clarifying instructions to facilitators, changes in terminology) often led to Refinements across all sessions and curriculums.

After the final session, outcome data were collected from pilot test participants at both sites via focus groups and surveys to gauge acceptability, appropriateness, and feasibility [27]. All pilot participants (youth and caregivers) were invited to participate in data collection. Participants provided informed consent and one minor participant provided assent with parent providing consent. Pilot focus groups were conducted virtually to accommodate COVID-19 protocols. Pilot feedback, as well as a comprehensive review of the adapted curriculum by project leadership, was incorporated into the final product.

## Results

### Phase 1: Assessment

Pre-adaptation focus group interviews were transcribed verbatim and transcripts were analyzed by a qualitative analysis team composed of three research assistants (RAs; two undergraduates RAs and one graduate RA) mentored by a Ph.D. researcher (project director) with extensive experience in qualitative methodology. All RAs had at least one year of research experience, were familiar with the TBRI curriculum, and received training in qualitative coding in Atlas.ti from the project director. The team adopted a deductive, consensus coding approach wherein the three research assistants independently coded the answers to specific prompts on TBRI content (e.g., TBRI terminology), delivery, and relevant topics (e.g., youth reentry, family environments; see Table 2 for examples of quotations and respective contexts). The three RAs discussed discrepancies and reached consensus on coding to achieve 100% coding agreement, with any coding disagreement resolved by the project director. Information was pooled across sites to inform the adaptation, with specific nuances for individual sites noted.

Qualitative analysis of the focus groups revealed individual and contextual characteristics of youth and families and provided information regarding caregiver backgrounds, family structures/compositions, the reentry process following discharge, the degree to which caregivers were involved in discharge planning, caregiver work schedules, physical distances between homes and JJ sites, and other logistical factors. For example, participants reported that when caregivers live within an hour of the facility, evening sessions are more feasible, whereas weekend sessions are preferred when caregivers must travel more than one hour. Such information was used to create a series of caregiver modules that could be delivered one at a time or bundled together, enabling the content to remain consistent but allowing flexibility in delivery across sites.

### Phase 2: Decision

Decisions regarding what and how to revise the curriculum were guided by core elements of the TBRI model: (1) teach participants about trauma and how to view behavior through a trauma-informed lens; (2) train participants to use TBRI’s three principles of Connecting, Empowering, and Correcting to foster healthy relationships, promote felt-safety, and improve regulation abilities; (3) provide opportunities for skills practice; and (4) model TBRI through all interactions with participants [12]. It is important to note that core elements of this EBI were not only about the content that is taught but how the facilitator interacts with the participants (relationally, through skills practice and modeling). This is consistent with the content/structure approach to adaptation, but also with the overall spirit of TBRI as a relational intervention. Identifying these core elements ensured that the components necessary for intervention fidelity and effectiveness (i.e., the theorized mechanisms for change) remained intact while avoiding unnecessary rigidity regarding protocol adherence.

Feedback from focus groups and subsequent conversations in the assessment phase revealed needs to be addressed by the adaptation beyond the original adaptation targets listed above, including the need to (a) further revise content to be more appropriate for biological caregivers and avoid potential for feeling shamed, (b) provide youth-specific training modules to complement the training provided to caregivers in order to increase youth engagement and felt-safety, (c) help caregivers examine their own caregiving histories more deeply to increase motivation and readiness, (d) develop virtual option to increase caregiver access and participation (hybrid delivery model), and (e) develop a companion curriculum for youth/caregiver sessions to enable families to practice strategies and plan for discharge. Ultimately, the workgroup decided to adapt the original TBRI Caregiver Training into a four-component intervention package (described below).

### Phase 3: Prototype Development

The four components of the adapted TBRI intervention package include the *TBRI Caregiver Curriculum*, *TBRI Youth & Young Adult Curriculum, TBRI Nurture Groups for Justice-Involved Families*, and *TBRI Family Coaching Curriculum* (see Table 3). The first three components make up the *primary TBRI intervention* are intended for delivery while youth are in residential facilities. The *secondary TBRI intervention*, Family Coaching, provides in-home support following discharge. All curriculum components employ the core elements of TBRI described above. The four components were designed to be used in conjunction with each other as interrelated components of a comprehensive intervention package. However, to be mindful of potential accessibility issues in future distribution and a commitment to equity in access to services, each curriculum is self-contained. That is, facilitators using the *Family Coaching Curriculum* do not need to refer back to the *Caregiver Curriculum* or *Youth and Young Adult Curriculum* to successfully deliver sessions, nor are there prerequisites for youth or caregiver participation in any curriculum component. All curriculum components are accompanied by a standardized manual (facilitator guide or lesson plans) that describes how to deliver the intervention. For conceptual clarity, the various components of adaptation are presented in a matrix (see Table 4). Examples that highlight particular aspects of the adapted intervention are described below. In practice, however, individual adaptations are interrelated elements that do not function independently.

The TBRI Caregiver Curriculum. The *TBRI Caregiver Curriculum* retained 90% of the original content designed to teach caregivers about the impact of trauma, build self-reflection and relational capacity, and equip caregivers with tools to identify youth needs and provide appropriate support. The *Caregiver Curriculum* consists of an individual introductory module (1 hour) and 9 group caregiver-only modules (90 minutes each) delivered in 4 sessions. Curriculum materials include a Facilitator Guide and Caregiver Handbook. As the original EBI is a group skills training for caregivers, the LeSA Caregiver Curriculum did not require extensive adaptation. However, some adaptations were necessary to reflect differences in the target population. Adaptations to content included (a) modified discussion of attachment theory to emphasize adaptative strategies and reduce potential for shaming, (b) modified terminology to reflect change in target population from adoptive/resource parents to biological caregivers, and (c) modified tone to enhance sensitivity and inclusivity (e.g., changed ‘they’ to ‘we,’ removed phrase ‘children from hard places’). In addition, an introductory module called *Conversations* with *Caregivers*, was developed as a tool to build caregivers’ reflective capacity. The new module consists of a semi-structured interview between a caregiver and the intervention facilitator to establish rapport, explore the caregiver’s own parenting and attachment history in a private context, and encourage engagement with the program. The interview is derived from more comprehensive pre-training work required for professionals receiving TBRI Practitioner Training. Modifications to structure included (a) condensed training sessions to shorten overall program duration to accommodate facility/JJ system capacity and (b) added individual sessions for caregiver who were not ready/able to join group sessions.

The TBRI Youth & Young Adult Curriculum. Consistent with the *Caregiver Curriculum*, the *Youth Curriculum* is a group skills training designed to teach youth and young adults about the impact of trauma, build self-reflection and relational capacity, and equip youth with tools to promote their transition home. The Youth Curriculum was developed to ensure that youth have the same opportunities as their caregivers to learn about TBRI and practice new skills. The curriculum consists of eight sessions, each 45 minutes long. Curriculum materials include a Facilitator Guide and TBRI activity book (participant workbook). The curriculum covers the same key concepts, principles, and strategies as the original Caregiver Training. However, notable adaptations were made to both content and structure to fit JJ facilities/systems and address the needs of youth participants. In addition to modifications made to terminology and tone consistent with modifications made to the *Caregiver Curriculum*, adaptations to the content of the *Youth Curriculum* include (a) replaced Life Value Terms with activity in which youth create their own alternative terms, (b) replaced or modified activities to be age-appropriate, (c) replaced or modified activities for approved use in secure facilities, (d) introduced transition planning earlier to accommodate uncertain discharge dates, and (e) added activities that acknowledge youth/youth and youth/staff dynamics within facilities. Adaptations to structure included (a) condensed training sessions to shorten overall program duration to accommodate facility/JJ system capacity, (b) added participation guidelines for facility staff present during sessions, and (c) replaced participant workbook with TBRI activity book.

TBRI Nurture Groups for Justice-Involved Families. Nurture Groups are designed to immerse youth and caregivers in a safe, playful environment where they can practice social-emotional skills [28, 12]. Nurture Groups are integral to the original EBI and introduced to LeSA participants in the *Caregiver Curriculum* and the *Youth Curriculum*. In order to give youth and caregivers an opportunity to practice TBRI together, with shared opportunities to regulate, role-play new skills, and build healthy connections, four one-hour Nurture Groups were added to the LeSA curriculum. Each of the four Nurture Groups align with the topics taught during youth and caregiver training sessions and are designed to take place after *Youth Curriculum* sessions two, four, six, and eight. These stand-alone Nurture Groups follow the same session layout as the original Nurture Groups that accompany TBRI Caregiver Training. However, activities were adapted to accommodate the service setting, be age-appropriate for youth, and allow virtual delivery as needed. Nurture Group facilitators utilize a standardized set of lesson plans to guide the sessions, with options for alternate activities as needed.

The TBRI Family Coaching Curriculum. The final component of the intervention is designed to be delivered after youth transition out of JJ settings. *The Family Coaching Curriculum* provides in-the-moment support to youth and caregivers and reinforces and expands on the TBRI topics, skills, and tools taught during earlier training. Family Coaching utilizes a supportive interaction style to promote active participation and is designed to be applied in natural learning environments (e.g., at home), where the family can identify and practice skills that are important to them in their ‘real life’ [29]. Curriculum materials include a *Facilitator Guide* and individual *Reflection Journals* for both youth and caregivers. Adaptations to content are consistent with modifications to terminology and tone made to other curriculums. Each one-hour coaching session is structured to resemble a Nurture Group. Substantial adaptations to structure were needed to extend the original group training for caregivers to in-home sessions for individual families. Two coaching options were developed for the LeSA Project: a structured four-session coaching program and a responsive coaching program with no limit to session number [14]. The responsive coaching curriculum includes the same four sessions as structured coaching but offers six additional session topics as well as a customizable session template and activity bank to allow facilitators to deliver more sessions as requested.

### Phase 4: Testing and Integration

The adapted TBRI curricula were pilot tested with eight youth-caregiver dyads (one of which included a Spanish-speaking caregiver and a bilingual youth) from two post-adjudicated facilities. The delivery was conducted via virtual platform because of health-related restrictions due to the COVID-19 pandemic. The session completion rates for the *primary TBRI intervention* ranged from 81–100% across three intervention components; the completion rate for the *TBRI Family Coaching* was 67%. The primary reasons for not achieving a 100% completion rate were cancellation due to COVID-related restricted contact and movement and competing family priorities (e.g., no stable housing). Post-intervention interviews with youth, caregivers, and staff suggest that participants found the program feasible and acceptable. For example, one youth stated, “*I’m given voice and encouraged to speak up for my needs and learning how to communicate to my family about my needs when I go home.” Likewise, a caregiver said, “My son has opened up to me more where he had shut down and [TBRI facilitator] let him know, ‘Oh, you’re free to speak the way you feel, as long as you do it in the appropriate way*.” The site staff corroborated the feasibility and acceptability by stating “…*[E]specially during COVID, for the parents and the kids to be able to have that time to do the Nurture Groups together exponentially just aided in these kids’ treatment.*”

Theater testing revealed the opportunity to make additional adaptations to improve accessibility and retention. For example, the intervention was designed to support youth transitioning out of justice facilities, but discharge dates often change, meaning some youth continued to reside in justice facilities for an extended period after their TBRI training had ended. Booster sessions were added to give youth and caregivers monthly opportunities to review and practice TBRI skills. Further, although adaptations to accommodate virtual delivery were a necessary response to the COVID-19 pandemic, virtual options for caregivers also accommodate geographic barriers for caregiver participation (e.g., caregivers live far from JJ facility and/or have limited access to transportation) and continued to be available once restrictions to in-person delivery were lifted. Finally, caregiver materials were translated into Spanish to increase participant accessibility. Theater testing with a Spanish-speaking caregiver not only provided the opportunity to get feedback on the direct translation but revealed the need for further review to improve cultural equivalence. For example, direct translation of an activity called ‘coach vs. warden’ failed to capture the difference between providing youth with supportive, authoritative guidance (coach) and authoritarian control (warden), as the terminology was culture-specific. The facilitator and translator (who is a bilingual TBRI Practitioner) discussed the terminology, asked the Spanish-speaking participant for feedback, and alternative terms were adopted in the final curriculum.

## Discussion

The current study documented the systematic adaptation of TBRI Caregiver Training for youth in secure JJ facilities using the ADAPT-ITT framework. Adaptation began with assessment, during which critical information about the target population was gleaned from focus groups. Important insights included challenges youth face during their residential stay, the involvement (or lack thereof) of caregivers in the reentry planning process prior to discharge, and challenges within the family and community systems. This information provided important contextual information that informed content and structural adaptations.

During the second phase of adaptation, Decision, core intervention elements were identified to ensure fidelity to the model. For TBRI, these included changing participant mindsets to be more trauma-informed, experiential instruction on the three principles (*Connecting, Empowering, Correcting*), skill practice, and facilitator modeling TBRI in all interactions. Adaptations focused on how to deliver core elements in a way that was appropriate for all caregivers (i.e., biological, foster, adoptive, kinship), engaging for youth from diverse backgrounds, encouraged self-reflection with regard to attachment histories, utilized formats that could be delivered virtually if needed, and equipped both youth and caregivers with the same tools and information.

While the original Caregiver Training was designed as a parenting intervention, the team realized early in the adaptation process the value of training both caregivers and youth. Not only do youth have increasing roles, responsibilities, and influence within family systems compared to younger children but many YILS are parents themselves [30]. Therefore, training youth in TBRI could not only increase the effectiveness of intervention efforts within families, but also equip youth to learn effective parenting strategies themselves. Furthermore, to help prepare families to implement TBRI strategies at home after discharge, families needed opportunities to practice strategies together and to plan for their use at home. The resulting adaptation included Nurture Groups, whereby youth and caregivers met jointly after sessions two, four, six, and eight. Finally, because the transition home can be challenging for youth and caregivers and because it takes time to implement new practices within an existing family system, some families may benefit from coaching and support in the months following discharge. Therefore, four interrelated curriculum components were created that were designed to be used together: *TBRI Caregiver Curriculum, TBRI Youth & Young Adult Curriculum, TBRI Nurture Groups for Justice-Involved Families, and TBRI Family Coaching Curriculum*.

Aligned with the ADAPT-ITT model, the current study documented the importance of involving a multidisciplinary team in all aspects of the process when adapting interventions for a specific population or context. Soliciting input from multiple stakeholders is critical during early assessment and ensures that developers are aware of unique challenges and needs [24]. Involving participants through theater testing, and iteratively incorporating modifications in real time also increases the likelihood that the adapted intervention is appropriate and acceptable for the new target group [24]. Theater testing provided opportunities to test the adapted content and delivery modifications and make adjustments to material that was unclear or activities that were ineffective. Facilitators shared session feedback with the adaptation workgroup prior to delivery in a second pilot site, thus ensuring that the resulting content, activities, and structure were appropriate and relevant.

Pilot feedback suggests that youth actively engaged in session activities and discussion, both in their youth-only groups and in joint sessions with their caregivers. Caregivers also actively participated and requested that sessions continue after the intervention conclusion. Caregivers also noted that as a result of TBRI, youth were more capable of expressing their needs, and caregivers felt better equipped to meet those needs. JJ staff also responded favorably, stating that the TBRI intervention was feasible and acceptable, and that they observed positive behavioral changes among youth who participated. Staff emphasized the value of TBRI for increasing family support and involvement during the reentry process, corroborating literature emphasizing the importance of family involvement in substance prevention [31, 32]. Further, data collected as part of adaptation efforts document improvement in outcomes among eight youth that participated in theater testing. Results (reported elsewhere) demonstrate that improvement occurred in youth impulsivity, conduct problems, hyperactivity, substance use, and the quality of youth-caregiver relationships from pre- to post-intervention [27].

## Limitations

The current study has limitations that should be noted. First, although the adaptation process was informed by multiple stakeholders, including members of the target population and practitioners with experience implementing the intervention in JJ systems, many of these individuals had existing relationships with TBRI and the research team and may therefore represent a biased sample. Furthermore, youth and caregiver perspectives were not solicited directly during the assessment phase. Instead, JJ staff spoke on their behalf (e.g., introducing elements of the curriculum to youth and reporting back to the research team). These issues, as well as the relatively small number of individuals participating in focus groups and pilot sessions, may limit generalizability to the larger population of justice-involved youth and families and/or JJ settings.

## Implications And Future Directions

Although researchers and practitioners are increasingly encouraged to adapt evidence-based models, only recently has there been an attempt to provide guidance for adaptation [33]. While there is growing acknowledgment that ad hoc adaptation is already common in practice and thus important to address in implementation frameworks, most of the literature on adaptation is conceptual or theoretical in nature [34]. The field of implementation science stands to benefit from studies that move beyond a conceptual framework for adaptation to include the why, what, and how adaptations are executed in practice, including details on the specific tasks, challenges, and solutions as well as the practical tools and documented processes that can inform future implementation [22]. In addition, this systematic approach of adapting an intervention for a better fit helps address the “tension” between scientific adherence of intervention protocols and cultural and content adaptation to meet needs of stakeholders [35].

Data demonstrating the initial acceptability and feasibility of the adapted TBRI curricula is a critical first step in efforts to establish evidence of the effectiveness of TBRI for JJ youth. The goal of the LeSA project is to improve SU and related outcomes for justice-involved youth participating in a trauma-informed intervention with their caregivers. While early feedback suggests that caregivers and youth found the intervention to be helpful, research is needed to establish the effectiveness of the adapted intervention for improving self-regulation and relationships with caregivers and preventing opioid and other substance use. A delayed-start, randomized control trial is currently underway to examine the added value of TBRI compared to standard reentry practice, the differential effectiveness of three post-discharge TBRI support formats, and the costs associated with TBRI implementation [14].

## Conclusions

The US JJ system has great potential to deliver quality SU prevention intervention at scale and with significant reach [9]. Yet the unique needs of JJ youth (e.g., high rates of trauma exposure) make it di cult to implement existing interventions without significant modifications. Investing time and resources to proactively plan and execute curriculum adaptation can improve the fit of existing EBIs for JJ settings, promote buy-in among stakeholders (including youth, caregivers, staff), and foster sustainability while encouraging fidelity to the model. The current project provides an example of how a planned, purposeful adaptation process involving the collaborative efforts among researchers, program developers, content experts, and practitioners can be incorporated into implementation projects.

## Figures and Tables

**Table 1:** Summary of Focus Group Interviews Conducted for Needs Assessment

Site	Interview Length (hours)	Number of Participants	Participant Titles
*Interviews on JJ-Youth Reentry and Family Environment*			
1	2	7	Chief Juvenile Probation Officer; Assistant Chief Juvenile Probation Officer; Probation Supervision and Community Supervision; Juvenile Supervision Officer and Training Coordinator; Probation Officer; Case Managers (2)
2	2	2	Deputy Director of Programs at [State] Department of Juvenile Justice; Substance Use Prevention and Recovery Manager
2	1.5	3	Deputy Director of Programs at [State] Department of Juvenile Justice; Substance Use Prevention and Recovery Manager; Aftercare Specialist Supervisor
3	2	2	Director of Student Services; Medical Director
3	1	12	Director of Student Services; Program Director; Medical Director, Clinical Director; Case Managers (5), Therapists (3)
4	2	1	Program Manager
5	1.5	2	Placement Supervisor; Senior Caseworker Supervisor of specialized services
6	2.5	7	Assistant Executive Director, Director of Mental Health Services; Facility Administrator; Assistant Director of Mental Health Services; Director of Facilities; TBRI Coach and Mentor; Field Counselor Substance Use Counselor
*Interviews on TBRI Implementation in JJ Settings*			
1	2.5	3	Chief Juvenile Probation Officer; Assistant Chief Juvenile Probation Officer; case manager
7	2	7	Transformation Coordinator; Transformation Team members (3), Supervisor of Therapists, Shift Supervisor, Deputy Director of Residential Services
8	6	6	Director of Re-Entry Services, Youth Facility Superintendent; Manager of Research; Program Specialist Supervisor; Deputy Executive Director of Probation Services; Deputy Executive Director for State Services
6	2.5	7	Assistant Executive Director, Director of Mental Health Services; Facility Administrator; Assistant Director of Mental Health Services; Director of Facilities; TBRI Coach and Mentor; Field Counselor Substance Use Counselor

**Table 2:** Selected examples of TBRI Life Value Terms and Level of Responses

Themes	Quotations/Paraphrase	Contexts and illustrations
TBRI Life Value Terms (Selected)		
Authority	“Are you asking me or telling me, sir?”	The adolescent reversed roles, but the distinction between asking and telling is important
Offering choices	“You know what? We dropped the ball. We didn’t tell you. Can you make sure that when new girls come in that they know the three B rule so that they’re not caught off-guard? I’m sorry we didn’t get you up front.”	An officer noticed a girl wasn’t dressed according to code, so they made that girl in charge of helping everyone else. It gave her importance and agency
Offering choices	“You can come down right now, or you can come down in three minutes”	A kid was on the roof, and they used this approach to get him down. This is a good example of giving choices to adolescents
Respect	“Can you try that again?”	This is a simple way of asking for some respect
**Level of Responses**		
1. Playful Engagement	“Because if [youth name] would have had the ability to regulate himself, then he might not have pulled that trigger and gotten a double life sentence at 16 years old. And like just that, when talk that to the kids, they’re like- right?”	This quote emphasizes the impact that learning how to regulate can have on a child
2. Structured Engagement	“So the levels of engagement, I think, for staff helped them understand conceptually what does this look like > What does this mean?”	Learning the levels of engagement in a juvenile justice setting helps to humanize the kids
3. Calming Engagement	“…if you go to security, we’re going to have a special unit that two of our cities- or cities- two of our regulation units call the regulation zone, where there are calming spaces, a couple of holding spaces, in case you’re making threats, but you’re not being aggressive.”	This facility is going to provide a space that is meant to be a safe place for youth to deescalate and regulate themselves
4. Protective Engagement	“She was calm the entire time. She’s trying to be playful. She has her OC. And she said ‘I don’t want to spray you. Just put the chair down. I don’t want to do this. We don’t have to do this.’ She did everything that we were asking to do, and then we had a very over eager responding staff literally run around the corner and spray the kid.”	A youth was trying to break a window with a chair. One staff member was trying to deescalate her using the levels, and the other just jumped the gun. The situation was so much worse because that youth got sprayed when she was almost back to being regulated
5. Sensory Processing	“It feels safer and calmer in there than in any other dorm I’ve been in. And so the idea that that is actually regulating for the kids, I still think people maybe struggle that that’s a thing.”	They painted the dorms fun colors and put in rocking chairs to make it more home-like.
**TBRI Principles**		
Connecting	“…you’re going to establish yourself as a safe adult”	The idea of designating oneself as a safe adult could be useful in explaining the role of the adult in TBRI
Empowering	“We might go outside and scream at a tree, or find a crawdad, or whatever it takes, do some push-ups, do some kind of appropriate receptive activity; whatever that looks like- get a drink of water. But just to get you away from whatever you’re fixated on that has you so distracted, and allow you to get back online so that we can have a conversation about why you’re distracted.”	This story is about how one officer removes the escalated individual from the situation and allows them to get that anger and stress out in a healthy way so that they can then talk and try to understand what happened.
Correcting	“And so I would call your name, you gotta close your eyes. I would toss you the ball. In this exercise the ball represents your words. You can’t see it but you can feel it, and so we teach them about how words have power…”	The interviewee is describing a nurture group that they used to teach the youth to recognize and use their voice
**Others**	“I find teenagers give you the one-finger salute when you ask about the magic mustache a lot of times”	

**Table 3:** Final Adapted TBRI Intervention Package for Justice-Involved Youth and Families

Intervention Component	Format/Participants	Delivery (Session Duration)	Materials	Location
*Primary TBRI Intervention*				
*TBRI Caregiver Curriculum*	Group skills training/caregivers	10 modules [1 individual (1 hour), 9 group (90 min)] delivered over 4 sessions *Delivered concurrent with Youth & Young Adult Curriculum*	Facilitator Guide, Caregiver Handbook	Home (virtual) or JJ Facility
*TBRI Youth & Young Adult Curriculum*	Group skills training/youth	8 modules (45 min) *Delivered concurrent with Caregiver Curriculum*	Facilitator Guide, Youth Workbook	JJ Facility
*TBRI Nurture Groups*	Group skills practice/caregiver-youth dyads	4 sessions (1 hour) *Follows Youth & Young Adult Sessions 2,4, 6, & 8*	Nurture Group Lesson Plans	JJ Facility
*Secondary TBRI Intervention*				
*TBRI Family Coaching Curriculum*	In-home coaching/caregiver-youth dyads	2 core sessions (1 hour) with option for *Structured Coaching* (2 additional sessions) or *Responsive Coaching* (unlimited additional sessions as needed)	Facilitator Guide, Reflection Journal	Home

**Table 4:** Examples of Adaptations to TBRI Intervention

	Adaptations to Content	Adaptations to Structure
*New Contextual Factors*		
*Service Setting*	Added, removed, or modified activities for approved use in secure facilities^2,3^ Introduced transition planning earlier to accommodate uncertain discharge dates^2^ Added activities that acknowledge youth/youth and youth/staff dynamics within facilities^2^	Added monthly Nurture Group ‘booster sessions’ to accommodate extended discharge dates^3^ Shortened session length and overall training duration to accommodate facility schedules^1,2^ Added standardized participation guidelines for facility staff present during sessions^2,3^
*COVID-19 protocols*	Modified activities for virtual delivery^1,2,3,4^	Added standardized guidelines for virtual delivery in accordance with COVID-19 protocols^1,2,3,4^ Added strategies for building felt-safety in virtual sessions^1,2,3,4^ Offered technical support, including addition of ‘Module 0’ sessions to prepare participants for virtual training^,2,3,4^
*New Target Population*		
*Caregiver*s	Modified discussion of attachment theory to emphasize adaptive strategies and reduce potential for shaming^1,2^ Modified terminology to reflect change in population from adoptive/resource parents to biological caregivers^1,2,3,4^ Added 1:1 semi-structured interview between caregiver and facilitator as tool to build reflective capacity^1^ Developed translated participant materials to encourage participation among non-English-speaking caregivers^1^	Added individual sessions for caregiver who were not ready/able to join group sessions^1^ Included translator during sessions as needed^1^ Developed *Reflection Journal* to encourage participant self-reflection^4^
*Youth*	Replaced Life Value Terms with activity in which youth create their own alternative terms^2^ Added, removed, or modified activities to be age-appropriate^2,3^ Modified language for sensitivity and inclusivity (e.g., changed ‘triggers’ to ‘buttons,’ removed ‘children from hard places’)^1,2,3,4^ Modified tone (e.g., changed ‘they’ to ‘we’)^1,2,4^	Developed *Youth Workbook* to encourage active participation^2^ Developed *Reflection Journal* to encourage participant self-reflection^4^

1 Caregiver Curriculum,

2 Youth amp; Young Adult Curriculum,

3 Nurture Groups,

4 Family Coaching Curriculum

## Data Availability

The dataset (which includes individual transcripts) is not publicly available due to confidentiality policies.

## Abbreviations

ACEs: adverse childhood experiences
EBIs: evidence-based interventions
JJ: juvenile justice
LeSA: Leveraging Safe Adults
RA: research assistant
TBRI: Trust-based Relational Intervention
SU: substance use
YILS: youth in the legal system
